# Molecular evidence for the involvement of PPAR-δ and PPAR-γ in anti-inflammatory and neuroprotective activities of palmitoylethanolamide after spinal cord trauma

**DOI:** 10.1186/1742-2094-10-20

**Published:** 2013-02-01

**Authors:** Irene Paterniti, Daniela Impellizzeri, Rosalia Crupi, Rossana Morabito, Michela Campolo, Emanuela Esposito, Salvatore Cuzzocrea

**Affiliations:** 1 Department of Biological and Environmental Sciences, University of Messina, Viale Ferdinando Stagno D'Alcontres, Messina, 31-98166, Italy; 2 Manchester Biomedical Research Centre, Manchester Royal Infirmary, School of Medicine, University of Manchester, Manchester, M13 9WL, UK

**Keywords:** Peroxisome proliferator-activated receptor, Palmitoylethanolamide, Spinal cord injur

## Abstract

**Background:**

Palmitoylethanolamide (PEA) is an endogenous fatty acid amide displaying anti-inflammatory and analgesic actions. Moreover, several data have suggested that PEA reduced inflammation and tissue injury associated with spinal cord trauma and showed a regulatory role for peroxisome proliferator-activated receptor (PPAR)-α signaling in the neuroprotective effect of PEA. However, several other mechanisms could explain the anti-inflammatory and anti-hyperalgesic effects of PEA, including the activation of PPAR-δ and PPAR-γ. The aim of the present study was to carefully investigate the exact contribution of PPAR-δ and PPAR-γ in addition to PPAR-α, in the protective effect of PEA on secondary inflammatory damage associated with an experimental model of spinal cord injury (SCI).

**Methods:**

SCI was induced in mice through a spinal cord compression by the application of vascular clips (force of 24 g) to the dura via a four-level T5 to T8 laminectomy, and PEA (10 mg/kg, intraperitoneally, 1 and 6 hours after SCI) was injected into wildtype mice and into mice lacking PPAR-α (PPAR-αKO). To deepen the ability of specific PPAR-δ and PPAR-γ antagonists to reverse the effect of PEA, mice were administered GSK0660 or GW9662, 30 minutes before PEA injection.

**Results:**

Genetic ablation of PPAR-α in mice exacerbated spinal cord damage, while PEA-induced neuroprotection seemed be abolished in PPARαKO mice. Twenty-four hours after spinal cord damage, immunohistological and biochemical studies were performed on spinal cord tissue. Our results indicate that PPAR-δ and PPAR-γ also mediated the protection induced by PEA. In particular, PEA was less effective in PPAR-αKO, GSK0660-treated or GW9662-pretreated mice, as evaluated by the degree of spinal cord inflammation and tissue injury, neutrophil infiltration, proinflammmatory cytokine, inducible nitric oxide synthase expression and motor function. PEA is also able to restore PPAR-δ and PPAR-γ expression in spinal cord tissue.

**Conclusion:**

This study indicates that PPAR-δ and PPAR-γ can also contribute to the anti-inflammatory activity of PEA in SCI.

## Background

Spinal cord injury (SCI) is the result of an initial physical trauma followed by a secondary degenerative process. SCI leads to the destruction of ascending and descending axonal tracts that control motor, sensory and autonomic functions. The level of SCI is an important factor because the consequences are devastating. Patients suffer from permanent, often lifelong motor and sensory disabilities below the site of injury, combined with impaired basic vital functions. Functional recovery is restricted because axons in the central nervous system (CNS) regenerate poorly. Post-traumatic inflammatory reaction may play an important role in the secondary injury processes after SCI [1,2]. In particular, the secondary damage is sustained by a large number of cellular, molecular, and biochemical cascades and a large body of data suggests the presence of a local inflammatory response, which amplifies the secondary damage [3].

Nuclear receptors are ligand-dependent transcription factors whose activation affects genes controlling vital processes. Among them, the peroxisome proliferator-activated receptors (PPARs) have emerged as links between lipids, metabolic diseases, and innate immunity [4]. PPARs are activated by fatty acids and their derivatives, many of which also signal through membrane receptors, thereby creating a lipid signaling network between the cell surface and the nucleus. There are three known isoforms of PPARs – PPAR-α, PPAR-γ, and PPAR-β/δ – each with different tissue specificity and physiological function [5]. All three isoforms share common molecular structure and functional domains similar to other nuclear receptor superfamilies. Upon ligand binding, PPARs forms a heterodimer with the retinoic acid receptor (RXR) and control the expression of genes that have PPAR response elements. PPARs exhibit complex ligand binding modes. PPAR C-terminal ligand binding domains are 60 to 70% homologous with large Y-shaped ligand binding pockets composed of three sub-arms that display significant homology between the subtypes. All three PPARs bind a variety of natural and synthetic ligands, none of which completely fills the ligand binding pocket, and PPAR ligands can adopt different binding modes. Many agonists, however, conform to a standard pharmacophoric model in which ligands comprise a hydrophilic head group that binds arm I and a hydrophobic tail that binds arm II and/or arm III [6]. PPAR-γ, or NRIC3, plays an important role in glucose and lipid homeostasis, inflammation, and adipocyte differentiation [7]. This transcription factor is further regulated by commonly known coactivator proteins such as CBP/p300, the SRC family, TRAP 220 [8], and co-repressors such as SMART, NCoR, and RIP140 [9]. The identification of an endogenous physiological ligand for PPAR-γ has been problematic. Most of the studies determining the binding efficiency have been performed in either cell-free or cell-based systems. The specificity of compounds to act as ligands for PPAR-γ was determined by a lack of response when cells were either pretreated with a known antagonist of PPAR-γ or with constructs that lacked PPAR ligand binding domain. However, in cell-based systems it is conceivable that a metabolite of the parent compound, not the parent compound itself, might be mediating the response through interactions with PPAR-γ. PPAR-δ activation improves the overall metabolic profile [10]. While no PPAR-δ agonists are yet approved for human use, they have been shown to enhance fatty acid oxidation in skeletal muscle, reduce serum triglycerides, increase serum high-density lipoprotein cholesterol and stimulate aspects of reverse cholesterol transport, improve glucose homeostasis, and trigger thermogenesis and weight loss [11].

Several studies have clearly demonstrated that PPAR-α and PPAR-γ agonists exert beneficial effects in several experimental models of CNS injury and disease, such as amyotrophic lateral sclerosis [12,13], Parkinson’s disease [14,15], cerebral ischemia or hemorrhage [16,17], and experimental autoimmune encephalomyelitis [18,19]. Recently, the presence of PPAR-α and PPAR-γ in discrete areas of the brain and spinal cord has been suggested [20,21].

Indeed, we have recently demonstrated using PPAR-α knockout (PPAR-αKO) mice that endogenous PPAR-α activity reduces the degree of development of inflammation and tissue injury events associated with spinal cord trauma in mice, suggesting the existence of an intrinsic anti-inflammatory mechanism mediated by PPAR-α [22]. In addition, an endogenous ligand for PPAR-γ, the cyclopentanone prostaglandin 15-deoxy-Δ^12,14^PGJ_2_, which is a metabolite of the prostaglandin D_2_, reduces the development of inflammation and tissue injury associated with spinal cord trauma [22].

*In vivo* administration of the saturated *N*-acylethanolamine palmitoylethanolamide (PEA), which explicates several effects on CNS such as modulating the pentobarbital-evoked hypnotic effect [23], controlling inflammatory pain [24], protecting against 1-methyl-4-phenyl-1,2,3,6-tetrahydropyridine (MPTP)-induced neurotoxicity [25] and learning and memory impairment in mice [26], reduces inflammation and tissue injury associated with SCI [27] and promotes the initiation of neurotrophic substance after SCI [28]. While the biological effects of PEA are well documented, the molecular mechanisms and site of action remain under debate. *In vitro*, PEA behaves as an endogenous agonist for a cannabinoid receptor (CB)_2_-like receptor subtype on mast cells [29], can inhibit gap junction formation, and modulates GABA and 5-HT receptors. *In vivo*, the analgesic effect of PEA is reversed by the CB_2_ antagonist SR144528 [30], while PPAR-α mediates the anti-inflammatory effects of PEA in carrageenan-induced paw edema and phorbol ester-induced ear edema, suggesting that this fatty-acid ethanolamide may serve, like its analog OEA, as an endogenous ligand for PPAR-α [31]. There is, as yet, little direct evidence for possible interactions of PEA with PPAR-δ or PPAR-γ in the spinal cord [32]. Our study will investigate whether the protective and anti-inflammatory effects of PEA observed in a compression model of SCI are partially mediated by other PPAR isotypes, in addition to PPAR-α. In fact, while we have recently demonstrated that PPAR-α modulates the anti-inflammatory property of PEA in a mouse model of inflammatory pain [33], on the contrary Benetti and colleagues have shown that CB_1_, PPAR-γ and TRPV1 receptors mediated the antinociception induced by PEA in mice with chronic constriction injury of the sciatic nerve [10].

To characterize the role of PPAR-α, PPAR-γ and PPAR-δ in PEA-mediated anti-inflammatory and neuroprotective activities, we performed spinal cord trauma in mice and studied the involvement of all of PPAR isotypes in mediating acute effects of PEA on the spinal cord.

## Methods

### Animals

Mice (6 to 7 weeks old, 20 to 27 g) with a targeted disruption of the PPAR-α gene (PPAR-αKO) and littermate wildtype controls (PPAR-α WT) were purchased from Jackson Laboratories (Harlan Nossan, Italy). Mice homozygous for the Ppara^tm1Gonz^ targeted mutation (strain name: 129S4/SvJae-Ppara^tm1Gonz^/J) are viable, fertile and appear normal in appearance and behavior. The study was approved by the University of Messina Animal Care Review Board. The animals were housed in a controlled environment and provided with standard rodent chow and water. Animal care was in compliance with regulations in Italy (D.M. 116192), Europe (O.J. of E.C. L 358/1 12/18/1986), and USA (Animal Welfare Assurance No A5594-01, U.S. Department of Health and Human Services, Washington, D.C USA.

### Surgical procedures

Mice were anesthetized with sodium pentobarbital (50 mg/kg, intraperitoneally). A longitudinal incision was made on the midline of the back, exposing the paravertebral muscles. These muscles were dissected away exposing T5 to T8 vertebrae. The spinal cord was exposed and SCI was produced by extradural compression of the spinal cord at T6 to T7 using an aneurysm clip with a closing force of 24 g as previously described [22]. Following surgery, 1 ml saline was administered subcutaneously in order to replace the blood volume lost during surgery. During the surgery and the recovery from anesthesia, the mice were placed on a warm heating pad and covered with a warm towel. The mice were singly housed in a temperature-controlled room at 27°C for a survival period of 10 days. Food and water were provided to the mice *ad libitum.* During this time period, the animals' bladders were manually voided twice a day until the mice were able to regain normal bladder function. In all injured groups, the spinal cord was compressed for 1 minute. Sham-injured animals were only subjected to laminectomy. Spinal cord tissues were taken at 24 hours after trauma. Tissue segments contained the lesion (1 cm on each side of the lesion), according to counts of retrogradely labeled neurons at the origin of distinct descending motor pathways and to morphometric assessments of normal residual tissue at the injury epicenter, as previously described [28].

WT mice were randomly allocated into the following groups: sham+vehicle group, mice were subjected to the surgical procedures as for the above group except that the aneurysm clip was not applied and vehicle was administered at 1 and 6 hours after laminectomy (*n* = 30); sham+PEA group, identical to sham+vehicle group except for the administration of PEA at 1 and 6 hours after laminectomy (*n* = 30); sham+ GW9662 group, identical to sham+PEA group except for the administration of GW9662 (1 mg/kg intraperitoneal bolus) 30 minutes prior to vehicle (*n* = 30); SCI+vehicle group, mice were subjected to SCI (*n* = 30); SCI+PEA group, PEA (10 mg/kg) was given daily as an intraperitoneal injection at 1 and 6 hours after SCI (*n* = 30); SCI+GSK0660, identical to the SCI+vehicle group but GSK0660 was administered (1 mg/kg intraperitoneally) 30 minutes and 5 and 0.5 hours and after SCI (*n* = 30); and SCI+GSK0660+PEA group, identical to the SCI+PEA group but GSK0660 was administered (1 mg/kg intraperitoneally) 30 minutes prior to PEA.

PPAR-αKO mice were randomly allocated into the following groups: PPAR-αKO sham group, PPAR-αKO mice were subjected to the surgical procedures as for the above group except that the aneurysm clip was not applied and vehicle was administered at 1 and 6 hours after laminectomy (*n* =30); PPAR-αKO+PEA group, identical to the PPAR-αKO sham group except for the administration of PEA 1 and 6 hours after laminectomy (*n* =30); PPAR-αKO SCI+vehicle group, PPAR-αKO mice were subjected to SCI and vehicle was administered at 1 and 6 hours after laminectomy (*n* =30); PPAR-αKO SCI+GW9662 group, identical to the PPAR-αKO SCI+vehicle group but GW9662 was administered (1 mg/kg intraperitoneal bolus) 30 minutes and 5 and 0.5 hours after SCI (*n* =30); PPAR-αKO SCI + PEA group, PPAR-αKO mice subjected to SCI received PEA injection (10 mg/kg) at 1 and 6 hours after SCI (*n* =30); and PPAR-αKO SCI+GW9662+PEA group, identical to the PPAR-αKO SCI+PEA group but GW9662 was administered (1 mg/kg intraperitoneally) 30 minutes prior to PEA.

PEA was purchased from Tocris Bioscience (Bristol United Kingdom, UK), dissolved in ethanol:saline (1:9), and used at a dose of 10 mg/kg based on a previous *in vivo* study [26].

GSK0660 and GW9662 were purchased from Sigma-Aldrich (Milan, Italy) and dissolved in a 1:1:8 mixture of ethanol:Tween80:saline. The dose of GW9662 (1 mg/kg, intraperitoneally) and GSK0660 (1 mg/kg, intraperitoneally) used here was based on previous studies [34,35].

### Tissue processing and histology

At the indicated time point (24 hours), spinal cords were dissected and tissue segments containing the lesion (1 cm on each side of the lesion) were paraffin embedded and cut into sections 5 μm thick. The tissue segments were fixed for 24 hours in paraformaldehyde solution (4% in PBS 0.1 M) at room temperature, dehydrated by graded ethanol and embedded in Paraplast (Sherwood Medical, Mahwah, NJ, USA). Tissue sections were deparaffinized with xylene, stained with H & E and studied using light microscopy connected to an imaging system (AxioVision; Zeiss, Milan, Italy). All of the histological studies were performed in a blinded fashion. The segments of each spinal cord at T6 to T7 vertebrae levels were evaluated by an experienced histopathologist. The histopathological changes of the gray matter were scored on a six-point scale: 0, no lesion observed, 1, gray matter contained one to five eosinophilic neurons; 2, gray matter contained 5 to 10 eosinophilic neurons; 3, gray matter contained more than 10 eosinophilic neurons; 4, small infarction (less than one-third of the gray matter area); 5, moderate infarction (one-third to one-half of the gray matter area); and 6, large infarction (more than one-half of the gray matter area). The scores from all sections from each spinal cord were averaged to give a final score for individual mice. All of the histological studies were performed in a blinded fashion.

### Myeloperoxidase activity

Myeloperoxidase (MPO) activity, an indicator of polymorphonuclear leukocyte accumulation, was determined as previously described [36]. At the specified time following SCI, spinal cord tissues were obtained and weighed, and each piece was homogenized in a solution containing 0.5% (w/v) hexadecyltrimethyl-ammonium bromide dissolved in 10 mM potassium phosphate buffer (pH 7) and centrifuged for 30 minutes at 20,000 × *g* at 4°C. An aliquot of the supernatant was then allowed to react with a solution of tetramethylbenzidine (1.6 mM) and 0.1 mM hydrogen peroxide. The rate of change in absorbance was measured spectrophotometrically at 650 nm. MPO activity was defined as the quantity of enzyme degrading 1 μmol peroxide/minute at 37°C and was expressed in milliunits per gram of wet tissue.

### Grading of motor disturbance

All animals were gentled prior to injury so that they were comfortable being handled and walking in the open field apparatus. Using the Basso Mouse Scale (BMS) locomotor rating scale [37], mice were tested prior to injury to ensure that all animals began with a normal score of 21. Every day post injury the mice were then scored for 4 minutes by two blind observers for all the treatment groups. Scores for each hindlimb were averaged, and then used to create group means at each day. Groups were compared using two-way repeated-measures analysis of variance followed by Bonferroni *post-hoc* analysis.

### Measurement of TNFα and IL-1β concentration

Perilesional tissue, collected 24 hours after SCI, was homogenized as previously described in PBS containing 2 mmol/l phenylmethylsulfonyl fluoride (PMSF) (Sigma Chemical Co. Milan, Italy and tissue TNFα and IL-1β levels were evaluated. The assay was carried out by using an ELISA colorimetric kit (eBioscience San Diego, CA USA according to the manufacturer’s instructions. All TNFα and IL-1β determinations were performed in duplicate serial dilutions.

### Western blot analysis

Spinal cord tissues, obtained from mice 24 hours after SCI or vehicle injection, were disrupted by homogenization on ice in lysis buffer (HEPES 20 mm, pH 7.9; 420 mm NaCl; 1.5 mm MgCl_2_; 1 mM ethylene glycol-bis(β-aminoethyl ether)-*N*,*N*,*N*^′^,*N*^′^-tetraacetic acid; 0.2 mM ethylenediamine tetraacetic acid; 25% (vol/vol) glycerol; 0.5% Nonidet P-40; 0.5 mM PMSF; 1.5 μg/ml trypsin inhibitor; 3 μg/ml pepstatin A; 2 μg/ml leupeptin; 0.1 mM benzamidine; and 0.5 mM dithiothreitol). After 1 hour, tissue total lysates were obtained by centrifugation at 100,000 × *g* for 15 minutes at 4°C. Nuclear extracts were prepared in extraction Buffer A containing 0.2 mM PMSF, 0.15 μM pepstatin A, 20 μM leupeptin, 1 mM sodium orthovanadate, homogenized at the highest setting for 2 minutes, and centrifuged at 1,000 × *g* for 10 minutes at 4°C. The pellets, containing enriched nuclei, were re-suspended in Buffer B containing 1% Triton X-100, 150 mM NaCl, 10 mM Tris–HCl pH 7.4, 1 mM ethylene glycol-bis (β-aminoethyl ether)-*N*,*N*,*N*^′^,*N*^′^-tetraacetic acid, 1 mM ethylenediamine tetraacetic acid, 0.2 mM PMSF, 20 μM leupeptin, 0.2 mM sodium orthovanadate. After centrifugation for 30 minutes at 15,000 × *g* at 4°C, the supernatants containing the nuclear protein were stored at −80°C for quantifying nuclear expression of PPAR-γ and PPAR-δ. The protein concentrations were estimated by the Bio-Rad protein assay (Bio-Rad Laboratories, Segrate, Milan, Italy) using BSA as standard. Seventy micrograms of proteins were dissolved in Laemmli’s sample buffer, boiled for 5 minutes, and subjected to SDS-PAGE (8% polyacrylamide). The blot was performed by transferring proteins from a slab gel to nitrocellulose membrane at 240 mA for 40 minutes at room temperature using TRANS-BLOT® SD Semy-dry Transfer Cell (BIO-RAD Laboratories Milan, Italy ). The filters were blocked with 1× PBS, 5% (w/v) nonfat dried milk for 40 minutes at room temperature and subsequently probed with specific antibodies anti-inducible nitric oxide synthase (anti- iNOS, 1:2,000; Transduction Laboratories, California USA), PPAR-γ (1:500; Novus Biologicals, Littleton, CO, USA), and PPAR-δ (1:500; Santa Cruz Biotechnology, Santa Cruz, CA, USA ) in 1× PBS, 5% w/v nonfat dried milk, 0.1% Tween-20 at 4°C, overnight. Membranes were incubated with peroxidase-conjugated bovine anti-mouse IgG secondary antibody (1:5,000; Jackson ImmunoResearch, West Grove, PA, USA) for 1 hour at room temperature.

To ascertain that blots were loaded with equal amounts of protein lysates, they were also incubated in the presence of the antibody against β-actin or lamin (1:10,000; Sigma-Aldrich Corp.). Signals were detected with enhanced chemiluminescence detection system reagent according to the manufacturer’s instructions (SuperSignal West Pico Chemiluminescent Substrate; Pierce Thermo Scientific Rockford, IL USA). The relative expression of the protein bands of iNOS (~125 kDa), PPAR-γ (~55 kDa), and PPAR-δ (~52 kDa) was quantified by densitometry with ImageQuant TL software (GE Healthcare Milan Italy and standardized for densitometric analysis to housekeeping gene levels. Images of blot signals were imported to analysis software (Image Quant TL, v2003; Sunnydale, CA, USA). A preparation of commercially available molecular weight markers (Precision Plus Protein Kaleidoscope standards; BIO-RAD) consisting of proteins of molecular weight 10 to 250 kDa was used to define molecular weight positions and as reference concentrations for each molecular weight.

### Statistical analysis

All values in the figures and text are expressed as the mean ± standard error of the mean of *n* observations. For the *in vivo* studies, *n* represents the number of animals studied. In the experiments involving histology, the figures shown are representative of at least three experiments performed on different experimental days. The results were analyzed by one-way analysis of variance followed by a Bonferroni *post-hoc* test for multiple comparisons. *P* <0.05 was considered significant. BMS scale data were analyzed by the Mann–Whitney test and considered significant when *P* <0.05.

## Results and discussion

### Role of functional PPAR-α gene in the protective and anti-inflammatory properties of PEA on the degree of spinal cord trauma

**Figure 1:**
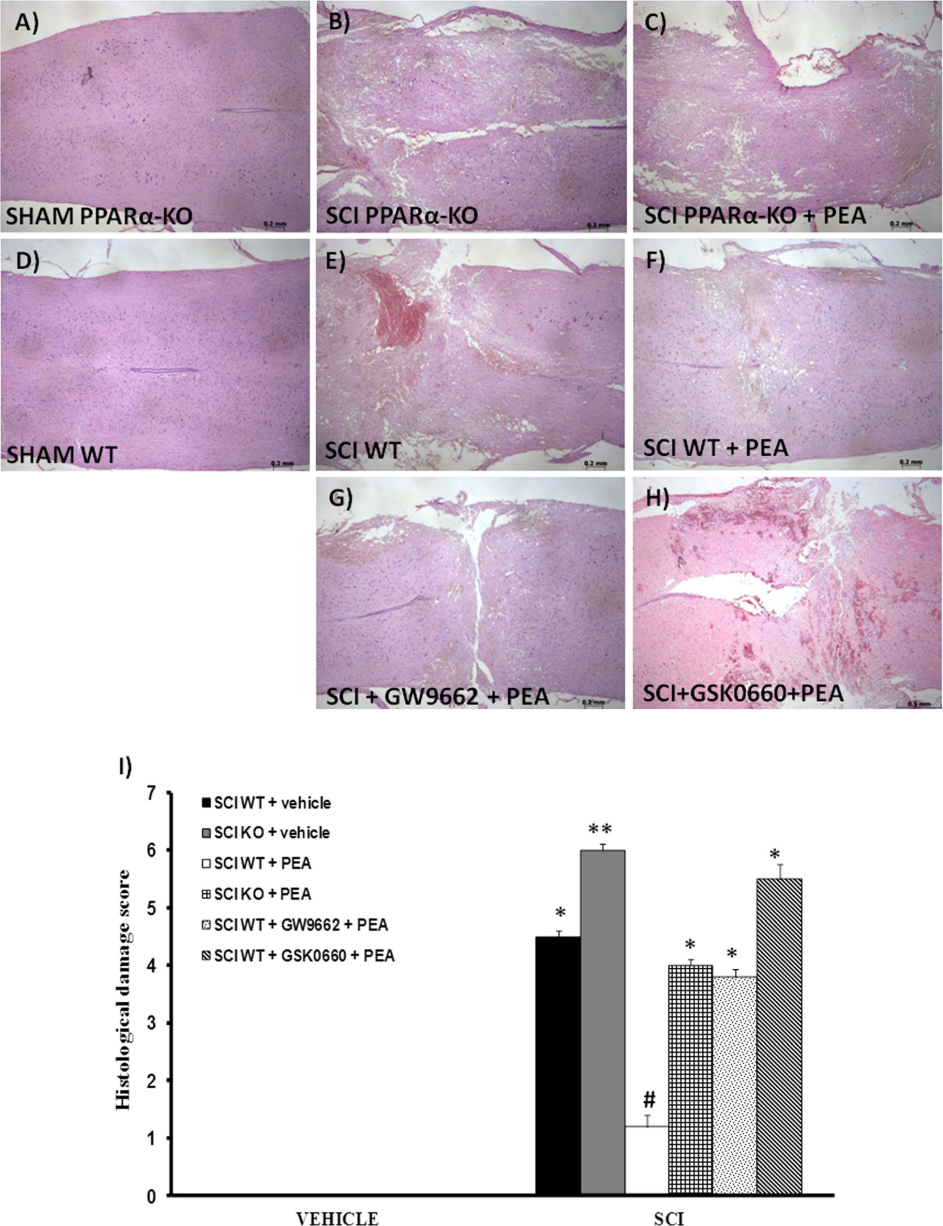
**Involvement of peroxisome proliferator**-**activated receptors on the protective actions of PEA following spinal cord trauma.** Following spinal cord compression a significant damage to the spinal cord from wildtype (WT) mice (**E**) at the perilesional zone was observed by **H** &**E** staining when compared with spinal cord tissue collected from the sham group (**D**). The absence of the peroxisome proliferator-activated receptor (PPAR)-α gene significantly increases the extent and severity of the histological damage (**B**) when compared with the sham group (**A**). Treatment with palmitoylethanolamide (PEA, 10 mg/kg) resulted in a significant decrease in the extent and severity of damage (**F**). The genetic absence of the PPAR-α receptor significantly blocked the effect of the PEA treatment (**C**). Pretreatment with GW9662 (1 mg/kg (**G**)) or GSK0660 (1 mg/kg (**H**)) counteracted the actions of PEA. The histological score (**I**) was made by an independent observer. These figures are representative of at least three experiments performed on different experimental days. Data are mean ± standard error of the mean of 10 mice for each group. *P <0.05 vs. sham WT; **P <0.01 vs. sham WT; #*P* <0.05 vs. spinal cord injury (SCI) WT.

As PPAR-α is constitutively expressed in astrocytes and neurons, it has been proposed that PPAR-α regulates brain and spinal cord lipid homeostasis during physiological conditions. PPAR-α activation may improve fatty acid mobilization, supporting both functional modification and structural reorganization in the dorsal horn of the spinal cord, such as activation of the arachidonate cascade, or axon sprouting. Spinal cords from non-injured mice appeared normal by gross and microscopic examination (Figure 1A,D). Twenty-four hours after the trauma, intra-parenchymal hemorrhages and cell swelling were visible throughout the white matter of injured cords (Figure 1B,E, see histological score I), but not in sham-operated mice. We found widespread damage to the spinal cord in H & E-stained sections taken through the centre of injury at T6 to T7 in PPAR-αΚΟ mice (Figure 1B,I). PPAR-α deletion increased spinal cord edema, which is a well-recognized cause of secondary neuronal damage after SCI in humans and animals. The genetic absence of the PPAR-α in KO mice significantly reduced the effect of the PEA treatment (Figure 1C,I).

### Involvement of PPAR-δ and PPAR-γ in the protective effects of PEA during SCI

PEA post-treatment (10 mg/kg) limited SCI-induced cytotoxic edema and infiltration of inflammatory cells (Figure 1F,I). Co-administration of GW9662 (1 mg/kg), a potent PPAR-γ antagonist, attenuated PEA-induced protection observed after SCI (Figure 1G,I). Similarly, co-administration of GSK0660 (1 mg/kg), a PPAR-δ antagonist, blocked the effect of PEA post-treatment on severity of damage.

### Role of PPAR-α, PPAR-δ, and PPAR-γ in PEA-induced inhibition of polymorphonuclear neutrophil infiltration

**Figure 2:**
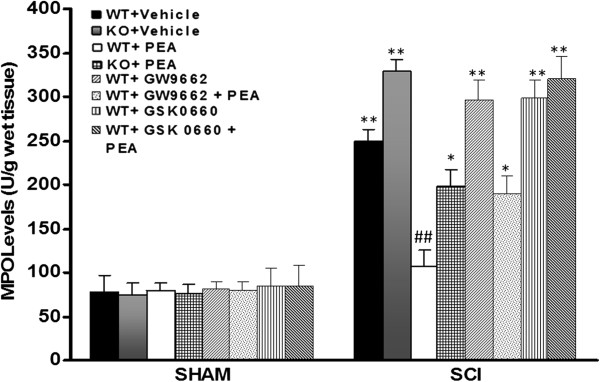
**Effect of PPAR-γ and PPAR-δ antagonist on the palmitoylethanolamide-induced decrease of myeloperoxidase activity.** Myeloperoxidase (MPO) activity was significantly increased in spinal cord injury (SCI) wildtype (WT) mice in comparison with respective control. Treatment with palmitoylethanolamide (PEA, 1 and 6 hours after injury) significantly reduced the neutrophil influx. The genetic absence of the peroxisome proliferator-activated receptor (PPAR)-α receptor significantly increased the activity of this peroxidase and blocked the effect of PEA. Pretreatment with GSK0660 (1 mg/kg) or GW9662 (1 mg/kg) neutralized the effect of PEA. Data are mean ± standard error of the mean of 10 mice for each group. *P <0.05 vs*.* WT sham; **P <0.01 vs*.* WT sham; #*P* <0.05 vs*.* SCI WT group.

The histological pattern of SCI appeared to correlate with the influx of leukocytes into the spinal cord. The results of the assay for the quantization of neutrophil influx by MPO activity shown in Figure 2 revealed that this peroxidase was increased in the spinal cord (T6 to T8 tract) from SCI mice 24 hours after the injury. Determination of spinal cord MPO revealed higher levels in the injured spinal cord from PPAR-αKO mice 1 day post lesion as compared with the WT SCI group. On the contrary, treatment with PEA (1 and 6 hours after SCI) resulted in a significant inhibition of the upregulation of MPO activity. The genetic absence of the PPAR-α significantly reduced the effect of the PEA on the neutrophil influx following experimental compression-type spinal cord trauma. Moreover, co-administration of potent PPAR-γ antagonist also significantly reduced the effect of the PEA treatment on MPO (Figure 2). The PPAR-δ receptor antagonist, GSK0660, was able to block the effect of PEA on MPO activity. All of the antagonists employed, when administered alone to SCI mice, did not affect this parameter.

### Role of PPAR-α, PPAR-δ, and PPAR-γ in PEA-induced inhibition of spinal TNFα and IL-1β

**Figure 3:**
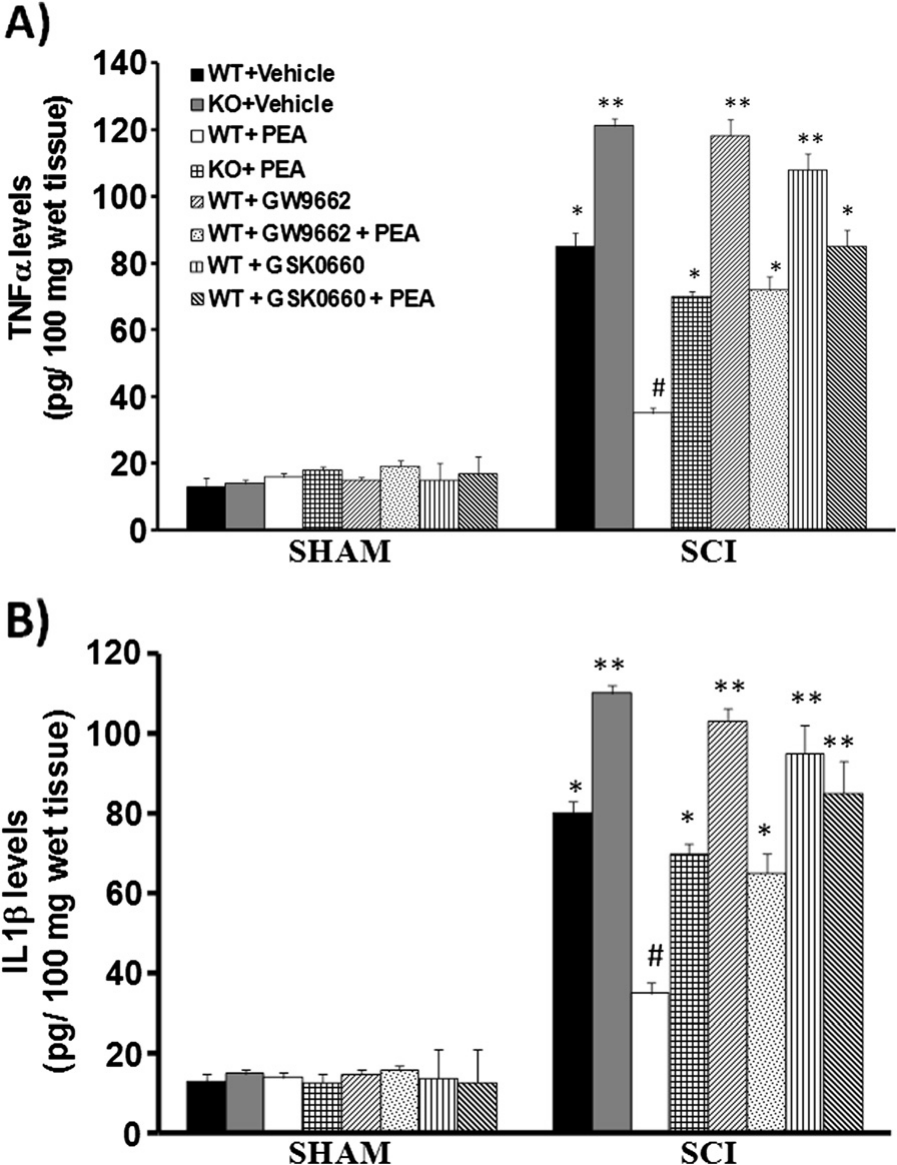
**Role of peroxisome proliferator-activated receptors in palmitoylethanolamide-induced TNFα and IL-1β inhibition after spinal cord trauma.** Wildtype (WT) mice showed a significant production of cytokines 24 hours after spinal cord compression (**A**), (**B**). Cytokine levels were enhanced in peroxisome proliferator-activated receptor (PPAR)-αKO mice when compared with the spinal cord injury (SCI) WT group. Treatment with palmitoylethanolamide (PEA, 10 mg/kg) significantly reduced the spinal cord TNFα (A) and IL-1β (B) production. The genetic absence of the PPAR-α receptor, as well as GSK0660 or GW9662 pretreatment, significantly blocked this reduction. Data are mean ± standard error of the mean of 10 mice for each group. *P <0.05 vs*.* sham WT; **P <0.01 vs*.* sham WT group; #*P* <0.05 vs*.* SCI WT group.

Proinflammatory cytokines present in the CNS could potentially regulate the expression of the CB_2_ receptor on microglial cells, astrocytes, and mast cells, whose expression level is likely to be important in the regulation of inflammation in the CNS during autoimmunity. Determination of TNFα and IL-1β levels by ELISA revealed high levels in the spinal cord (L6 to L7 tract) of SCI mice 24 hours post lesion as compared with sham animals (Figure 3A,B). Administration of PEA restored physiological TNFα and IL-1β levels (Figure 3). Spinal cord TNFα and IL-1β levels were significantly higher in PPAR-αΚΟ SCI mice in comparison with the WT SCI group (Figure 3). The genetic absence of the PPAR-α receptor significantly blocked the effect of the PEA on the production of proinflammatory cytokines. Similarly, either pretreatment with GSK0660, the specific PPAR-δ antagonist, or pretreatment with GW9662, the selective PPAR-γ receptor antagonist, reversed PEA-induced reduction of these cytokines.

### Involvement of all of three isotypes of PPAR in iNOS expression in the spinal cord

**Figure 4:**
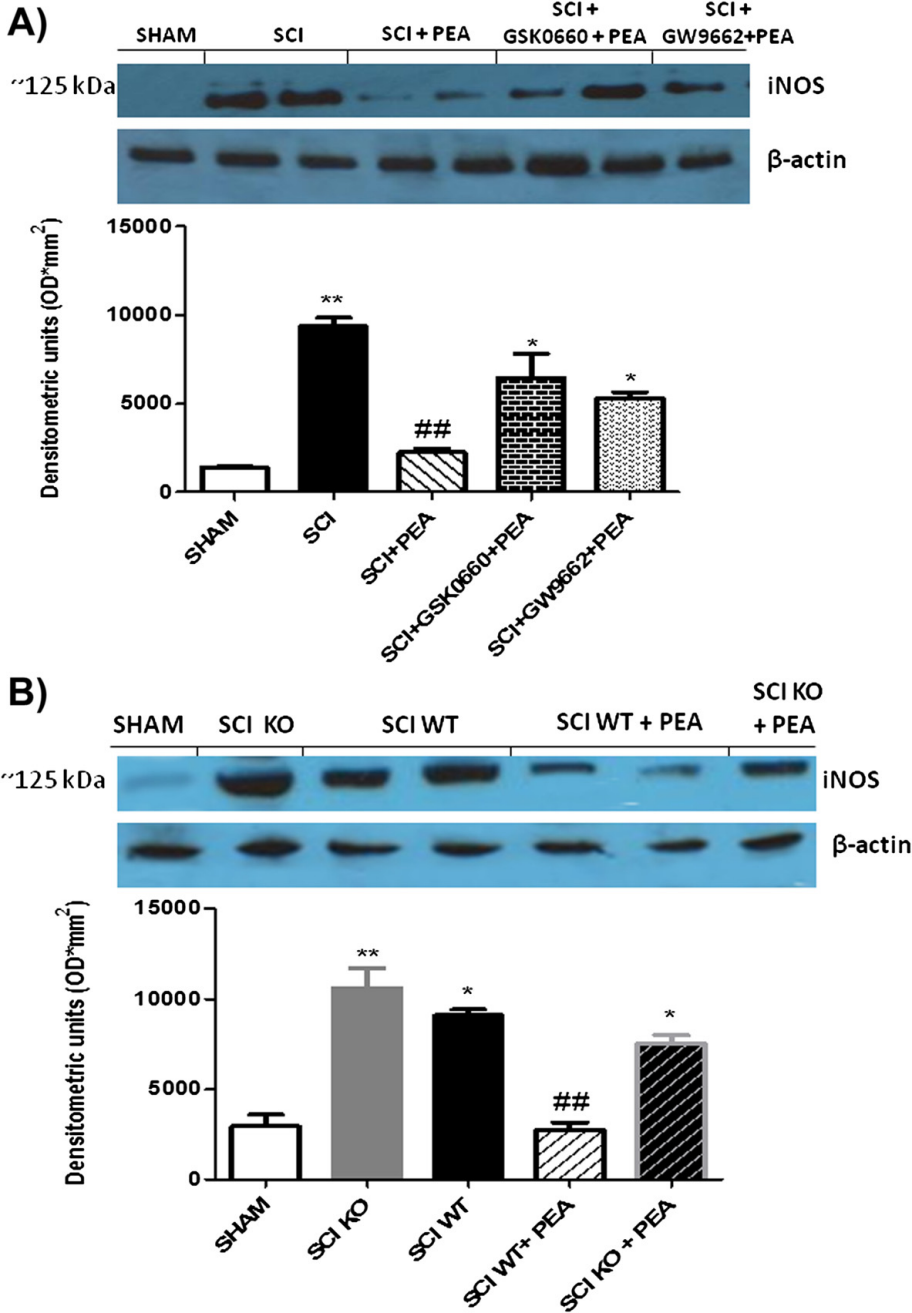
**Role of PPAR-α, PPAR-γ, and PPAR-δ in palmitoylethanolamide-induced inhibition of inducible nitric oxide synthase expression.** Spinal cord injury (SCI) induced an increase in inducible nitric oxide synthase (iNOS) expression evaluated by western blot analysis. Palmitoylethanolamide (PEA) post-treatment significantly reduced the levels of this protein in spinal cord homogenates. Either pretreatment with GW9662 (1 mg/kg) or GSK0660 (1mg/kg) counteracted the anti-inflammatory action of PEA (**A**). Mice with genetic absence of the peroxisome proliferator-activated receptor (PPAR)-α receptor exhibited increased expression of iNOS when compared with that observed in the SCI wildtype (WT) group and PEA was not able to reduce the expression of this enzyme in injured PPAR-αKO mice (**B**). *P <0.05 vs*.* sham WT; **P <0.01 vs*.* sham WT group; ##*P* <0.01 vs*.* SCI WT group.

iNOS expression is proposed to influence the outcome of the synaptic plasticity process in the spinal cord. Western blot analysis of the spinal cord using an iNOS-specific antibody showed that iNOS expression in mice tissues was increased after spinal cord compression (Figure 4A). PEA attenuated iNOS expression in injured WT mice (Figure 4), contributing to neuroprotection in a compressive model of SCI, while PEA was not able to reduce the expression of this NOS isozymes in the PPAR-αKO SCI group (Figure 4A). However, in PPAR-αKO SCI animals, levels of iNOS protein in the spinal cord were higher than that of WT SCI mice. Moreover, either pretreatment with GSK0660, the specific PPAR-δ antagonist, or pretreatment with GW9662, the selective PPAR-γ receptor antagonist, did reverse PEA-induced downregulation of iNOS (Figure 4B).

### Effects of PEA on spinal PPAR-γ and PPAR-δ expression

**Figure 5:**
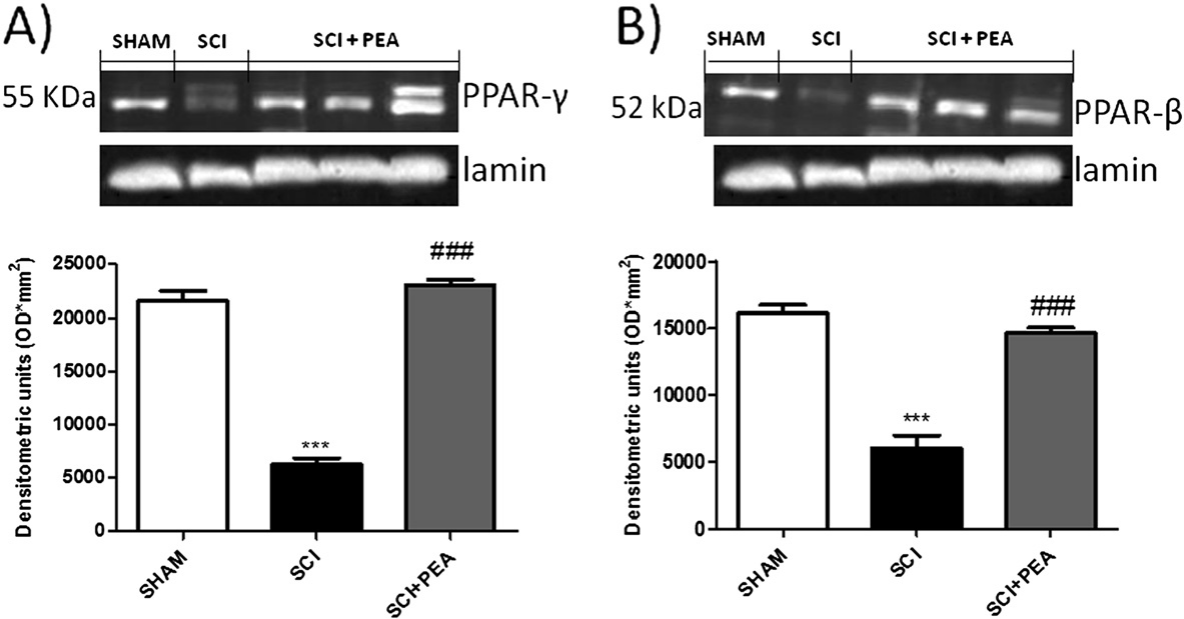
**Effect of palmitoylethanolamide-on PPAR-γ and PPAR-δ expression.** Spinal cord injury (SCI) induced a decrease of both PPAR-γ (**A**) and PPAR-δ (**B**) expression when compared with control group, evaluated by western blot analysis. Palmitoylethanolamide (PEA) post-treatment significantly restore the levels of both PPAR-γ (**A**) and PPAR-δ (**B**) in spinal cord homogenates. ***P <0.05 vs*.* sham; ###*P* <0.01 vs*.* SCI group.

PPAR-δ could participate to the transcriptional regulation of oligodendrocyte differentiation, and should regulate myelinogenesis by modulation of fatty acid metabolism. Western blot analysis shows that PPAR-γ (Figure 5A) and PPAR-δ (Figure 5B) were expressed in uninjured spinal cords (Figure 5). Twenty-four hours after SCI, PPAR-γ and PPAR-δ protein expression was significantly reduced in spinal cord homogenates. PEA treatment significantly restored the basal levels of spinal PPAR-γ and PPAR-δ.

### Role of PPAR-α, PPAR-δ, and PPAR-γ in PEA-induced reduction of motor dysfunction

**Figure 6:**
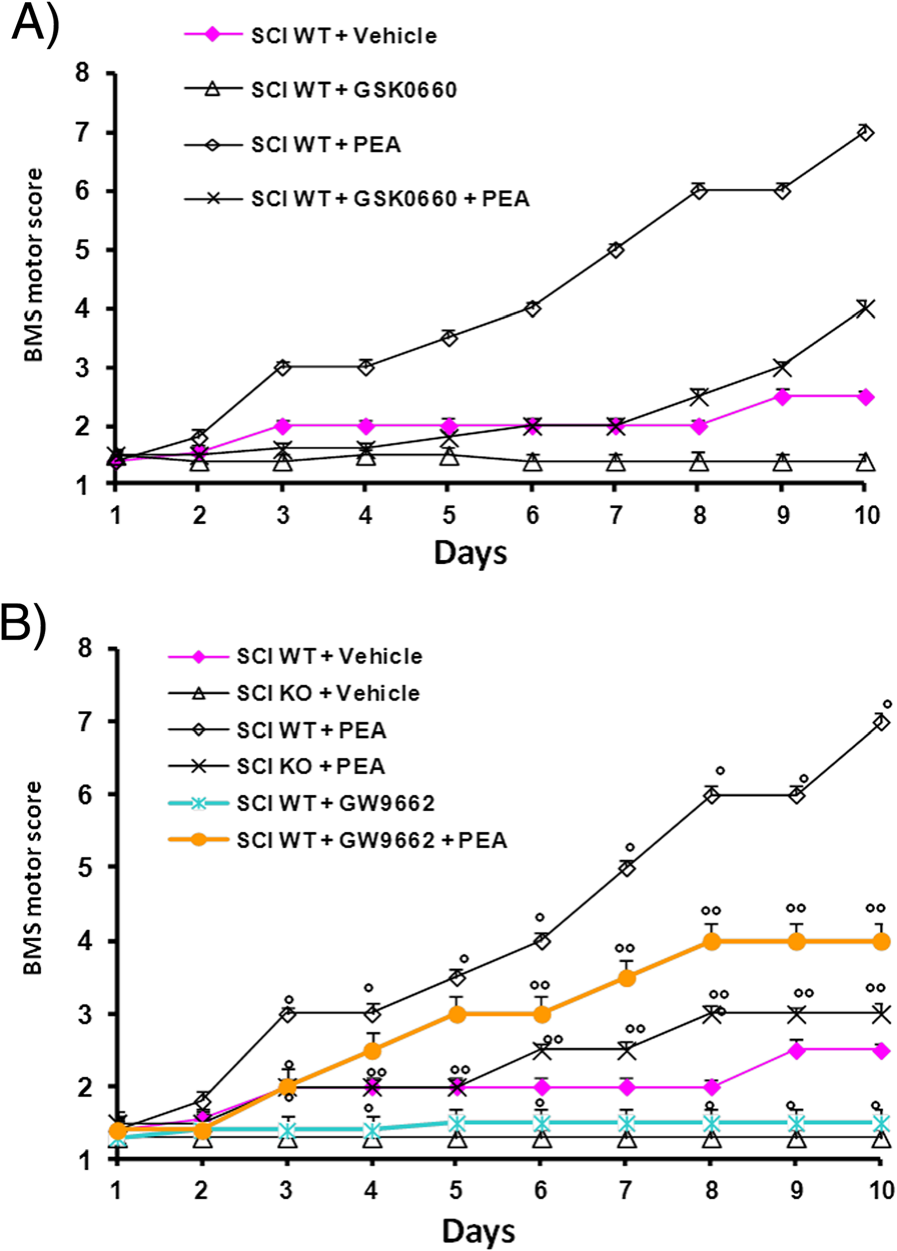
**Effects of GSK0660 and GW9662 treatment on palmitoylethanolamide-induced improvement of locomotion after spinal cord injury.** The degree of motor disturbance was assessed every day until 10 days after spinal cord injury (SCI) by Basso Mouse Scale (BMS) criteria. Palmitoylethanolamide (PEA)treatment (10 mg/kg) caused a significant increase in Basso, Beattie and Bresnahan (BBB) locomotor rating scale. Pretreatment with GSK0660 (**A**) or GW9662 (**B**) abolished PEA-induced improvement of locomotion after SCI. Overall BBB scores of injured PPAR-αKO mice (**B**) were lower than injured WT mice (**A**). PEA-treated PPAR-αKO mice did not show a recovery of locomotor function (**B**).

Traumatic SCI results in severe inflammation and decreased cellular regeneration, which lead to difficult functional recovery. Open field locomotor function was tested using the BMS starting 1 day post injury through the end of the study (10 days). While motor function was only slightly impaired in sham-operated mice, WT mice subjected to SCI displayed significant deficits in hind limb movement (Figure 6). In contrast, a significant worsened of hind limb motor disturbances was observed in the PPAR-αKO operated mice (Figure 6A). PEA treatment caused a significant increase in BMS scores (Figure 6A). The genetic absence of the PPAR-α receptor significantly blocked the effect of the PEA treatment (Figure 6A). Similarly, when we antagonized the PPAR-δ pathway using GSK0660, and PPAR-γ using GW9662, we found a worse locomotor function versus PEA group (Figure 6B).

## Conclusions

Our findings confirm that PPAR-α is involved in protective effects of PEA in spinal cord trauma. Moreover, the anti-inflammatory effect elicited by PEA was antagonized by the administration of the antagonists for PPAR-γ, and PPAR-δ receptors, highlighting the involvement of such receptors in PEA-induced protective effects of spinal cord trauma. PPARs are constitutively expressed in the lumbar and thoracic spinal cord of healthy animals. Activation of transcription factors is often an early molecular event preceding long-term changes in tissue function. While PPAR-α expression was weak in the gray matter, a high expression was observed in some cells in the white matter, especially in astrocytes. The presence of PPAR-α in astrocytes suggests that this isoform modulates central inflammation, possibly by regulation of cytokine production by astrocytes [38]. In the spinal white matter, both PPAR-α and PPAR-δ isoforms were expressed, but the cellular characterization using glial cells markers revealed their different cellular distribution. A high PPAR-δ expression was present in neurons, in the deep laminae of the dorsal horn, in the medial and central regions of the ventral horn, in the large motoneurons of the lamina IX and in the area X around the central canal of the spinal cord gray matter, in all cord segments, cervical, thoracic, or lumbar, studied. Moreover, PPAR-δ is present in oligodendrocytes, which are the major lipid producing cells in the nervous system and are responsible for myelination [39]. Several data have been reported showing that the anti-inflammatory effects of PEA result from its ability to activate PPAR-α-dependent gene transcription [31] and occur with a time lag of hours [40]. Often these slow-onset actions are preceded, however, by rapid antinociceptive responses whose time course is incompatible with a transcription-dependent process [41,42]. Even though PEA does not bind to PPAR-γ, its neuroprotective actions in SCI but also in controlled cortical impact [43] are prevented by the PPAR-γ antagonist GW9662. Moreover, we found that a PPAR-γ antagonist chemically unrelated to GW9662, the compound T0070907, also affected the response to PEA (data not shown). In addition to classical regulation of PPAR-γ activity by ligands, PPARγ transcriptional activity can be affected by post-translational modifications such as cross-talk with kinases and phosphatases and its interaction with other proteins in the cytoplasm that leads to nongenomic functions of PPAR-γ [44].

PPAR-α regulates systemic inflammation by inducing the expression of anti-inflammatory proteins, and repressing the expression of proinflammatory proteins, such as TNFα, and limiting the recruitment of immune cells to inflammation sites [45]. PPAR-α might regulate adaptive response to tissue injury by dampening inflammation through a slow-onset genomic mechanism. The first report of an anti-inflammatory activity of PEA was made by Coburn and colleagues in 1954 [46], but the anti-inflammatory effects of PEA may not be restricted to mast cells alone. PEA decreased the levels of the cytokine TNFα [47]. The same authors observed a similar inhibitory effect of PEA on IL-4, IL-6 and IL-8 release from human peripheral blood mononuclear cells [48]. More recently, Ross and colleagues reported that PEA reduced nitric oxide release from RAW264.7 macrophage cells in response to lipopolysaccharide by a CB-independent mechanism [49]. PEA has been shown to be effective in several experimental models of inflammation, both of immunogenic and neurogenic origin [40,50]. Despite its various described pharmacological properties, the cellular/receptor mechanism responsible for the actions of PEA is still debated. The first hypothesis on the mechanism of action of PEA was formulated when Aloe and colleagues [51], introducing the ALIA acronym (Autacoid Local Inflammation Antagonism), indicated that some endogenous *N*-acylethanolamines, such as PEA, exerted a local antagonism on inflammation [51]. Moreover, various studies have hypothesized that PEA may act via indirect interaction with CB_2_ receptors [30,52,53]. In fact, it has been shown that the use of SR144528, a CB_2_ specific receptor antagonist, eliminated the antinociceptive effects of PEA [41,50,54,55]. PEA was then found able to potentiate the effect of anandamide on CB or vanilloid receptor (VR1) [56,57], the so-called entourage effect. Considering the known changes of endocannabinoid system in the lesioned spinal cord (increase in 2-AG and CB_2_, decrease in CB_1_ levels), PEA may indirectly regulate CB receptors expression through the increase in endocannabinoid tone [43] or the modulation of proinflammatory cytokines. Indeed, CB_2_ receptors would play a crucial role in limiting the spreading of this neuroinflammatory process [58]. In the present study we show that the absence of PPAR-α, in PPAR-αKO mice, as well as the use of PPAR-δ and PPAR-γ antagonist, results in a reduced anti-inflammatory action of PEA in an experimental model of SCI. Part of our results are in agreement with previous observations indicating that PPAR-αKO mice are more susceptible to induction of SCI, possibly due to a less efficient anti-inflammatory control exerted by endogenous PPAR-α ligand [59-61]. But in this paper we show that also PPAR-δ and PPAR-γ can be involved in the neuroprotective and anti-inflammatory effects of PEA.

Recent evidence shows that PPAR agonists prevent neuronal damage and myelin loss other than pain behavior in a model of SCI [22,25,34,60]. The anti-inflammatory efficacy of PEA resides in part in the capability to counter NF-κB activation, the production of cytokines relevant to the inflammatory process including TNFα, the induction of iNOS and cyclooxygenase-2 as well the increase in their enzymatic activity [62,63].

Recently, Citraro and colleagues showed antiepileptic action of PEA through CB_1_ and PPAR-α receptor activation in a genetic model of absence epilepsy [64].

The results described here clearly indicate that the anti-inflammatory and protective efficacies of PEA treatment are favored by the presence of PPAR-α, PPAR-δ, and PPAR-γ.

The efficacy of PEA treatment in inflammatory and neurological diseases may become an important therapeutic target, and is necessary better understand the molecular mechanisms by which PEA therapy could be act. Results discussed here suggest a new mechanism contributing to determining the full PEA efficacy and suggest future studies should aim to analyze the possible relevance of PPAR-δ and PPAR-γ in other human inflammatory diseases. This suggests that PPAR-γ and PPAR-δ are also targets for PEA in protecting the spinal cord against proinflammatory insults. Since the actions of PEA were not through direct interaction with nuclear PPAR-γ and PPAR-δ, they are more probably mediated primarily by CB_1_-dependent changes in PPAR expression. In fact, PEA may also be able to restore neuroinflammation-induced downregulation of PPAR-γ expression through a CB_1_ receptor-dependent pathway. PEA binds to Gi-coupled CB_1_ receptors and/or CB_2_ receptors, and their activation suppresses phosphorylation of NF-κB through ERK/p38 mitogen-activated protein kinase and increases the expression of PPAR-γ, which represses inflammatory gene transcription [65,66].

## Abbreviations

BMS: Basso Mouse Scale; BSA: Bovine serum albumin; CB: Cannabinoid receptor; CNS: Central nervous system; ELISA: Enzyme-linked immunosorbent assay; H & E: Hematoxylin and eosin; IL: Interleukin; KO: Knockout; iNOS: Inducible nitric oxide synthase; MPO: Myeloperoxidase; NF: Nuclear factor; PBS: Phosphate-buffered saline; PEA: *N*-acylethanolamine palmitoylethanolamide; PMSF: Phenylmethylsulfonyl fluoride; PPAR: Peroxisome proliferator-activated receptors; SCI: Spinal cord injury; TNF: Tumor necrosis factor; WT: Wildtype.

## Competing interests

The authors declare that they have no competing interests.

## Authors’ contributions

IP and DI carried out the *in vivo* experiments, and drafted the manuscript. RM carried out the immunoassays and performed statistical analysis. MC participated in the western blot analysis. RC performed immunohistochemical analysis. EE and SC conceived of the study, and participated in its design and coordination and helped to draft the manuscript. All authors read and approved the final manuscript.
